# Emergency Department Presentation of Duloxetine-induced Acute Extrapyramidal Symptoms: A Case Report

**DOI:** 10.5811/cpcem.56947

**Published:** 2026-07-10

**Authors:** Michael W. Shulby, Christina M. Powell

**Affiliations:** * Naval Medical Center Portsmouth Department of Emergency Medicine, Portsmouth, Virginia; † Naval Medical Center Camp Lejeune, Department of Emergency Medicine, Camp Lejeune, North Carolina

**Keywords:** duloxetine, extrapyramidal symptoms, acute dystonia, drug-induced movement disorder, case report

## Abstract

**Introduction:**

Duloxetine, a serotonin-norepinephrine reuptake inhibitor, has been associated with extrapyramidal symptoms and tardive syndromes; however, such adverse reactions are rare and remain sparsely documented, particularly in the setting of non-psychiatric use.

**Case Report:**

A young, healthy, active-duty military service member developed acute extrapyramidal symptoms—restlessness, dystonic movements, and acute dystonia—shortly after initiating duloxetine for postsurgical neuropathic pain.

**Conclusion:**

This case highlights the need for vigilance regarding movement disorders in patients prescribed duloxetine, even in the absence of psychiatric comorbidity or antipsychotic exposure.

## INTRODUCTION

Extrapyramidal symptoms encompass a spectrum of drug-induced movement disorders, including akathisia, dystonia, parkinsonism, and tardive dyskinesia. These symptoms are most associated with dopamine receptor blocking agents, particularly antipsychotics, due to their antagonism of dopaminergic pathways in the basal ganglia.^1^–^3^ Although antipsychotics remain the leading cause, antidepressants have also been implicated in the development of extrapyramidal symptoms. Mechanistically, serotonergic antidepressants may disrupt dopaminergic transmission, predisposing susceptible individuals to movement disorders.^4^–^6^ This case highlights the unusual occurrence of acute extrapyramidal symptoms—including akathisia and dystonic movements—in a healthy, young, active-duty military service member following recent initiation of duloxetine for postsurgical neuropathic pain.

## CASE REPORT

A 23-year-old active-duty U.S. Marine presented to the Naval Medical Center Emergency Department (ED) with a four-hour history of progressively worsening restlessness and involuntary muscle movements. The patient had been initiated on duloxetine 30 mg daily one week prior for management of postsurgical lower extremity neuropathic pain. His last dose of duloxetine had been taken approximately five hours before symptom onset, and adherence to the prescribed regimen was confirmed. The only other daily medication the patient reported was celecoxib for his lower extremity pain.

Upon arrival, the patient’s vital signs were within normal limits. Physical examination demonstrated acute dystonia with prominent psychomotor agitation, characterized by abnormal facial grimacing, blepharospasm, and dystonic movements of the neck and limbs. No other focal neurologic deficits were appreciated. Electrocardiography and routine laboratory studies were unremarkable. Initial management with intravenous diphenhydramine did not improve the patient’s symptoms. Subsequent administration of oral benztropine resulted in complete and rapid resolution of the extrapyramidal manifestations.

The patient was discharged from the ED with a prescription for two additional doses of benztropine, to be taken only if symptoms recurred. He was advised to discontinue duloxetine and adhere to close outpatient follow-up. At a one-week follow-up appointment with his primary care physician, the patient reported complete and sustained resolution of symptoms following cessation of duloxetine.

## DISCUSSION

Duloxetine is widely prescribed for depression, anxiety, and chronic pain. The adverse event profile is generally dominated by gastrointestinal and central nervous system effects, with movement disorders considered rare. Although antidepressants are less commonly implicated, serotonin–norepinephrine reuptake inhibitors such as duloxetine have been reported to induce extrapyramidal symptoms through secondary effects on dopaminergic neurotransmission.^6^–^7^ Excessive serotonergic activity can indirectly suppress dopaminergic transmission within the basal ganglia via inhibitory serotonergic pathways. This mechanism may precipitate acute dystonic or dyskinetic reactions, particularly in susceptible individuals or in the setting of rapid dose initiation or titration. In this case, the temporal relationship between duloxetine initiation and symptom onset supports a causal association.

Large pharmacoepidemiologic studies and post-marketing surveillance have specifically identified duloxetine as one of the antidepressants with a measurable, although infrequent, association with extrapyramidal symptoms, with a rate ratio of 5.68 compared to controls.^4^ Case reports and U.S. Food and Drug Administration adverse event data further document isolated instances of duloxetine-induced dystonia and dyskinesia, often in patients without prior psychiatric or neurological comorbidities.^5^–^13^

From an emergency medicine perspective, recognition of medication-induced extrapyramidal symptoms is essential for prompt diagnosis and management. The differential diagnosis includes acute dystonic reaction, serotonin syndrome, neuroleptic malignant syndrome, and functional movement disorders. Key distinguishing features include the absence of hyperthermia, autonomic instability, or altered mental status, which effectively ruled out serotonin syndrome and neuroleptic malignant syndrome in this patient. Tardive dyskinesia was excluded based on the acute onset of symptoms, as this condition requires at least three months of exposure to dopamine receptor blocking agents and presents with movements that persist for at least four weeks, whereas acute dystonia develops within hours to days of medication initiation or dose increase.^14^

The mainstays of emergency management for acute extrapyramidal symptoms are prompt drug discontinuation and targeted pharmacologic therapy. While anticholinergic agents (eg, benztropine) are first-line treatment,^15^ it was not always readily available in the ED, prompting initial management with diphenhydramine. Anticholinergic agents restore dopaminergic–cholinergic balance within the basal ganglia. Benzodiazepines or propranolol may be used as adjuncts when needed.^15^ Importantly, the treatment approach differs fundamentally from tardive dyskinesia, where anticholinergics are ineffective and may worsen symptoms; instead, vesicular monoamine transporter-2 inhibitors (valbenazine, deutetrabenazine) are the recommended first-line therapy,^14^ underscoring the importance of accurate clinical diagnosis before initiating treatment.

*CPC-EM Capsule*

What do we already know about this clinical entity?

*Duloxetine and other serotonin–norepinephrine reuptake inhibitors rarely cause extrapyramidal symptoms via serotonergic inhibition of dopaminergic pathways.*

What makes this presentation of disease reportable?

*Acute dystonia and akathisia developed soon after duloxetine initiation for postsurgical neuropathic pain in a healthy young man without psychiatric history.*

What is the major learning point?

*Consider duloxetine as a potential cause of acute extrapyramidal symptoms, even in patients without antipsychotic exposure or psychiatric disease.*

How might this improve emergency medicine practice?

*Early recognition will help ensure timely treatment with anticholinergics.*

This case is notable for several distinguishing characteristics: the patient was an otherwise healthy military service member who developed acute dystonia after duloxetine initiation for post-surgical neuropathic pain—a non-psychiatric indication. This contrasts with the limited existing literature on duloxetine-induced movement disorders, which predominantly describes extrapyramidal symptoms in patients treated for psychiatric conditions such as major depressive disorder.^7^,^10^–^11^ Furthermore, while prior case reports have documented tardive syndromes developing after prolonged duloxetine exposure (18 months),^11^ acute dystonia occurring shortly after initiation in a young, healthy individual without psychiatric comorbidities has not been previously reported. The patient’s military status and lack of predisposing risk factors for movement disorders further underscore the clinical significance of this case.

Although duloxetine-induced extrapyramidal symptoms remain uncommon, clinicians should maintain vigilance when evaluating patients with new-onset movement disorders, particularly those recently initiated on serotonergic or noradrenergic antidepressants. Reporting such cases contributes to a broader understanding of atypical adverse drug reactions and reinforces the importance of medication review in emergency presentations of acute movement abnormalities.

## CONCLUSION

This case illustrates a unique presentation of extrapyramidal symptoms associated with recent outpatient initiation of serotonin–norepinephrine reuptake inhibitors for a non-psychiatric indication in an otherwise healthy, young, military service member. Given the potential morbidity associated with extrapyramidal symptoms and the widespread use of antidepressants, clinicians should maintain a high index of suspicion for drug-induced movement disorders in patients prescribed duloxetine. This case underscores the need for heightened clinical awareness of duloxetine-associated extrapyramidal symptoms in populations not typically considered at risk, such as young, physically active military personnel, and supports the importance of early recognition and intervention for these potentially distressing adverse effects.
